# Increased Susceptibility to *Plasmodium falciparum* in Infants is associated with Low, not High, Placental Malaria Parasitemia

**DOI:** 10.1038/s41598-017-18574-6

**Published:** 2018-01-09

**Authors:** Samuel Tassi Yunga, Genevieve G. Fouda, Grace Sama, Julia B. Ngu, Rose G. F. Leke, Diane W. Taylor

**Affiliations:** 1 Department of Tropical Medicine, Medical Microbiology and Pharmacology, John A. Burns School of Medicine, University of Hawaii at Manoa, 651 Ilalo Street, BSB320, Honolulu, HI 96813 USA; 2 Duke Human Vaccine Institute, Duke University Medical Center, Durham, North Carolina, USA and Department of Pediatrics, Duke University Medical Center, Durham, North Carolina USA; 3 The Biotechnology Center, University of Yaoundé 1, BP 3851 Messa, Yaoundé, Cameroon; 4 Faculty of Medicine and Biomedical Sciences, University of Yaoundé 1, BP 1364, Yaoundé, Cameroon

## Abstract

Risk of malaria in infants can be influenced by prenatal factors. In this study, the potential for placental parasitemia at delivery in predicting susceptibility of infants to *Plasmodium falciparum* (Pf) infections was evaluated. Seventy-two newborns of mothers who were placental malaria negative (PM−) and of mothers who were PM+ with below (PM+ Lo) and above (PM + Hi) median placental parasitemia, were actively monitored during their first year of life. Median time to first PCR-detected Pf infection was shorter in PM + Lo infants (2.8 months) than in both PM− infants (4.0 months, p = 0.002) and PM + Hi infants (4.1 months, p = 0.01). Total number of new infections was also highest in the PM + Lo group. Only 24% of infants experienced clinical malaria episodes but these episodes occurred earlier in PM + Lo infants than in PM + Hi infants (p = 0.05). The adjusted hazard ratio (95% CI) of having Pf infection was 3.9 (1.8–8.4) and 1.5 (0.7–3.4) for infants in the PM + Lo and PM + Hi groups, respectively. Collectively, low placental parasitemia was associated with increased susceptibility to malaria during infancy. Therefore, malaria in pregnancy preventive regimens, such as sulfadoxine-pyremethamine, that reduce but do not eliminate placental Pf in areas of drug resistance may increase the risk of malaria in infants.

## Introduction

When pregnant women become infected with the mosquito-borne parasite *Plasmodium falciparum* (Pf), parasitized erythrocytes adhere to placental villi and accumulate in the intervillous spaces (IVS), causing a condition referred to as placental malaria (PM)^1^. In addition to eliciting adverse pregnancy outcomes^2^, PM has been identified as a risk factor for early childhood morbidity and mortality. In general, infants born to PM-positive (PM+) mothers have a shorter time to first Pf infection^3,4^ and first clinical episode of malaria^5,6^, a higher incidence of Pf infections early in life^7^, lower hemoglobin levels^8^, and higher risk of dying^9^ compared to infants of PM-negative (PM−) mothers. However, the risk of malaria is not homogeneous among infants of PM+ mothers, since some PM+ infants have similar risk outcomes as PM− infants^3,5,9–11^. There is therefore a need to identify specific prenatal factors associated with increased susceptibility to malaria in infants whose mothers had PM.

Factors thought to be important in modifying the susceptibility of infants to malaria include maternal gravidity and the timing of occurrence of malaria during pregnancy. For example, infants born to multigravid Tanzanian women had their first microscopically detected infection 12 weeks earlier than infants of primigravid women^3^. Likewise, Gabonese infants born to multigravidae were approximately twice as likely to experience clinical episodes of malaria during the first 30 months of life than infants born to primigravidae^5^. Concerning the timing of infection during pregnancy, when maternal Pf infections occurred within 3 months prior to delivery, Ugandan infants were at a higher risk of infection than when mothers were infected earlier in pregnancy^10^. It is possible that prenatal exposure to low levels of malarial antigens, rather than higher levels influences early childhood susceptibility since multigravidae tend to have lower parasite loads than primigravidae^12–14^ and women who are infected only late in pregnancy would be infected for a short time. No study has directly investigated the influence of placental parasitemia (parasite load in the placenta) on the risk of malaria in infants.

Therefore, the primary objective of this study was to determine if the susceptibility of Cameroonian infants to Pf was modified by maternal placental parasitemia at delivery. Susceptibility was measured by the median time to first post-natal infection or clinical malaria episode, hazard ratios and total number of times infants were infected during the first year of life. We hypothesized that infants born to mothers with low placental parasitemia (PM + Lo) would have shorter times to first Pf infection and experience more infections during the first year of life than infants whose mothers had higher parasitemias (PM + Hi) or whose mothers were negative for Pf (PM−). Secondarily, to determine if PM + Hi mothers experienced more infections and had higher peripheral parasitemia during the course of pregnancy, thereby potentially exposing their fetuses to higher levels of Pf antigens, maternal peripheral blood samples collected during the course pregnancy were also examined for Pf and umbilical cord blood samples at delivery were tested for Pf parasites and parasite products.

## Results

### Study population

**Figure 1:**
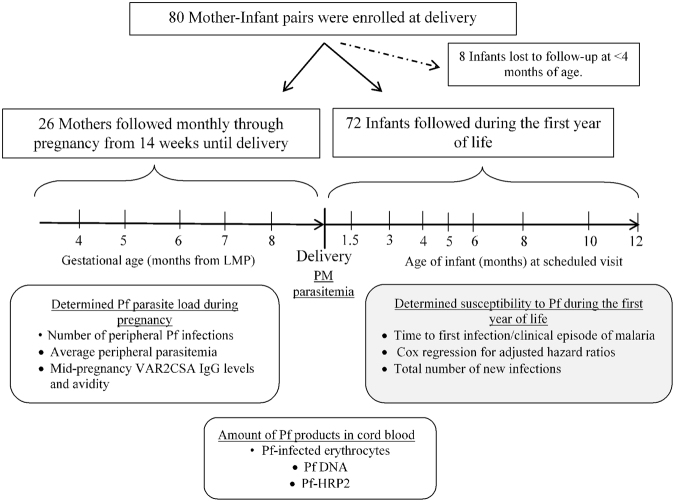
Study design. Placental *P*. *falciparum* (Pf) parasitemia at delivery was used to classify newborns into 3 groups, i.e., babies born to PM− (no placental Pf, n = 36), PM + Lo (<median placental parasitemia, n = 18), and PM + Hi (>median, n = 18) mothers. The 72 infants were actively monitored during their first year life to evaluate differences in susceptibility to Pf. Infant follow-up was conducted at scheduled visits and in-between if any illness was reported. To estimate differences in prenatal exposure to Pf of infants in the 3 study groups, peripheral blood samples collected monthly during pregnancy from mothers of 26 newborns were assessed for Pf infections and anti-PM antibodies, and umbilical cord blood samples of 70 newborns were tested for whole parasites, parasite DNA and histidine-rich protein 2 (HRP2). PM; placental malaria; LMP, last menstrual period.

Seventy-two infants (out of 80 initially enrolled at birth) that permanently resided in a high malaria-transmission village in Cameroon were followed longitudinally during their first year of life (Fig. 1). At delivery, mothers of half of the infants were PM+ (n = 36) with placental parasitemia ranging from 10,521 Pf-infected erythrocytes (IE)/µL (quantified in IVS blood smear) down to <14 IE/μL (i.e., only detectable by histology or impression smear), with a median parasitemia of 25 IE/µL. The distribution of placental parasitemia is shown in Supplementary Fig. S1. Infants born to PM+ mothers were sub-divided into PM + Lo (placental parasitemia < median; n = 18) and PM + Hi (placental parasitemia > median; n = 18). The average duration of post-natal follow-up was similar for all 3 groups of infants, i.e., PM− = 11.2 months [interquartile range = 8.2–12.2]; PM + Lo = 12.0 [11.4–12.4]; and PM + Hi = 12.0 [6.0–12.2]; p = 0.2. Thus, differences in malaria susceptibility among the groups were not influenced by duration of follow-up.

### Time from birth to first Pf infection

**Figure 2:**
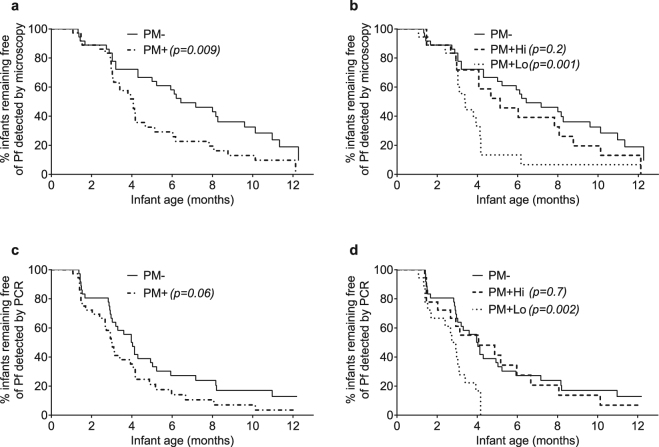
Kaplan-Meier curves showing time (in months after birth) that infants remained free of Pf infections detected by blood-smear microscopy (a and b) and PCR (**c** and **d**). The left panels (a and c) show results for infants born to PM− mothers (thin solid lines, n = 36) and PM+ mothers (thick dash-dotted lines, n = 36). In the right panels (b and d), infants born to PM+ mothers were further stratified into PM + Lo (dotted lines, n = 18) and PM + Hi (dashed lines, n = 18) groups. P values were obtained by log-rank test and evaluate differences in median time to first infection, between the PM+ groups and the PM− reference group.

*P*. *falciparum* infections were detected by microscopy and/or PCR at least once during the first year of life in 89% of infants (64/72). Overall, 55% (35/64) of first infections were detectable by microscopy. The median time to first microscopically detected infection was significantly shorter in infants born to PM+ mothers (4.1 months) compared to infants of PM−mothers (6.4 months), p = 0.009 (Fig. 2a). Further stratification showed that infants in the PM + Lo group had a significantly shorter time to first microscopic infection compared to infants in the PM− group (3.4 versus 6.4 months, respectively, p = 0.001). No significant difference in time to first microscopic infection was observed between infants in the PM + Hi and PM− groups (5.1 versus 6.4 months, p = 0.2) (Fig. 2b). A similar pattern was found for time to first infection detected by PCR (Fig. 2c,d), i.e., 2.8 months in the PM + Lo group compared to 4.0 months in the PM−(p = 0.002) and 4.1 months in the PM + Hi (p = 0.01) groups. By age 4 months, all babies in the PM + Lo group had experienced at least 1 Pf infection; whereas only 50% of infants in the other two groups had become infected (Fig. 2d). Taken together, infants in the PM + Lo group were more prone to being infected with Pf at an earlier age compared to infants in the other two groups.

### Time from birth to first clinical episode of malaria

The majority of first Pf infections were asymptomatic (92%, i.e., in 59 out 64 infants who were infected with Pf). Overall, 24% of all infants (17/72) experienced one or more clinical malaria episodes (fever plus blood-smear microscopy positive for Pf) during the first year of life, with 71% of episodes (12/17) occurring after 6 months of age. Because <50% of infants in each follow-up group had clinical malaria, the median time to first clinical episode was not calculated. However, there was a trend for clinical malaria to occur later during the first year of life in the PM + Hi group compared to the PM− group (p = 0.06) and the PM + Lo group (p = 0.05). By 10 months of age, only 5% of PM + Hi infants had experienced at least one clinical malaria episode compared to 22% of PM− infants and 40% of PM + Lo infants. Together, the results suggest that earlier episodes of clinical malaria were likely to occur in PM + Lo infants.

### Multivariate assessment of time to Pf infection

Using Cox regression, the effect of placental parasitemia at delivery on the time from birth to Pf infection was controlled for potential confounding factors including gravidity, climatic season when infants were born, hemoglobin genotype of infants, amount of transplacentally acquired anti-Pf merozoite antibodies in cord blood and zone of residence within the village (Table 1). The adjusted hazard ratio (HR) and 95% confidence interval (CI) for microscopically detected infections was 2.8 (1.3–6.0) in the PM + Lo group and 1.5 (0.7–3.1) in the PM + Hi group. Adjusted HR for infections detected by PCR was 3.9 (1.8–8.4) for PM + Lo infants and 1.5 (0.7–3.4) for PM + Hi infants. Therefore, the instantaneous risk of infection was significantly high in PM + Lo infants, and not in PM + Hi infants, compared to PM− infants. Multivariate Cox regression analysis was not performed for the outcome of clinical malaria episode because of the relatively small number of the clinical cases.

**Table 1:** Multivariate Cox regression analysis showing hazard ratios for Pf infections detected by microscopy and PCR during infancy. Hazard ratios (HR) were adjusted for all other variables on the table. *Zone A, central Ngali II village; Zone B, satellite Ngali II villages (Okoa, Abondo and Ntouessong); CI, confidence interval; Pf, *Plasmodium falciparum*; MSP1; merozoite surface protein 1; Hb, hemoglobin (HbAA, normal hemoglobin and HbAS, sickle cell trait). Bold fonts show HR and adjusted HR that are significantly higher in the corresponding subgroup than in the reference subgroup (i.e., lower limit of CI > 1.0).

Variable	Subgroup	% of 72 infants	Blood-smear microscopy		PCR	
			HR (95% CI)	Adjusted HR (95% CI)	HR (95% CI)	Adjusted HR (95% CI)
Placental parasitemia	High (PM + Hi)	24	1.5 (0.8–2.8)	1.5 (0.7–3.1)	1.1 (0.6–2.1)	1.5 (0.7–3.4)
	Low (PM + Lo)	26	**2**.**6 (1**.**3–4**.**8)**	**2**.**8 (1**.**3–6**.**0)**	**2**.**8 (1**.**5–5**.**4)**	**3**.**9 (1**.**8–8**.**4)**
	None (PM−)	50	Reference		Reference	
Residence*	Zone B	56	0.7 (0.4–1.2)	0.6 (0.3–1.2)	1.1 (0.6–1.7)	1.0 (0.6–1.9)
	Zone A	44	Reference		Reference	
Gravidity	Primigravid	14	1.3 (0.6–3.0)	1.0 (0.4–2.4)	1.0 (0.5–2.3)	0.9 (0.4–2.4)
	Multigravid	69	Reference		Reference	
Season at birth	Heavily rainy	15	0.6 (0.3–1.5)	0.3 (0.1–0.9)	0.7 (0.3–1.6)	0.5 (0.2–1.3)
	Dry	20	0.9 (0.4–1.9)	0.6 (0.2–1.7)	0.8 (0.4–1.7)	1.0 (0.3–2.9)
	Rainy	36	0.7 (0.4–1.3)	0.4 (0.2–1.0)	0.5 (0.3–1.0)	0.6 (0.2–1.2)
	Very dry	29	Reference		Reference	
Hb genotype	HbAS	25	1.0 (0.6–1.9)	1.4 (0.7–2.8)	1.2 (0.7–2.1)	1.3 (0.6–2.5)
	HbAA	75	Reference		Reference	
Cord MSP1 IgG level	Upper tertile	33	0.7 (0.4–1.5)	0.5 (0.2–1.3)	1.3 (0.7–2.4)	1.4 (0.6–3.3)
	Middle tertile	33	0.7 (0.3–1.4)	0.6 (0.3–1.2)	1.0 (0.5–1.9)	1.0 (0.5–2.1)
	Lower tertile	33	Reference		Reference	

### Total number of new infections infants had during the first year of life

**Figure 3:**
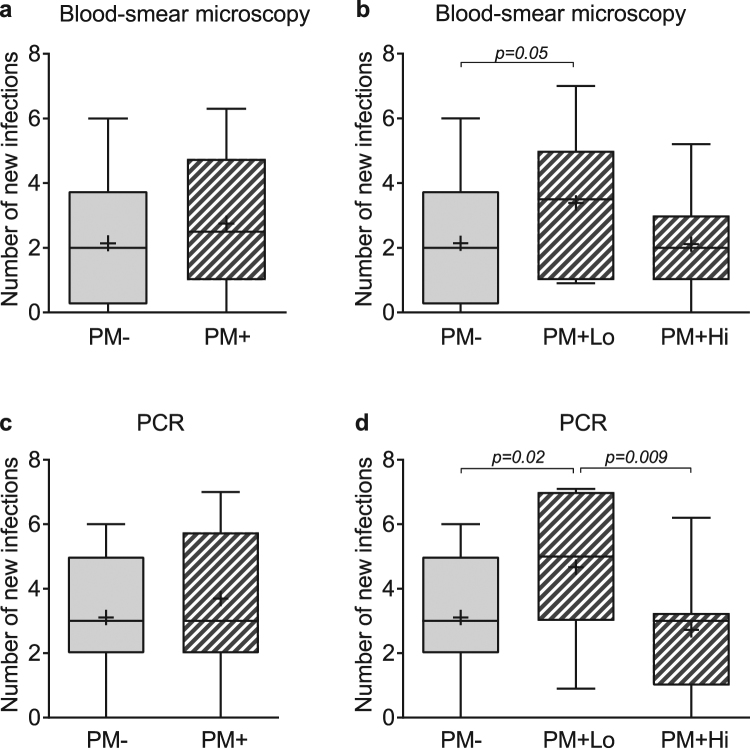
The number of new Pf infections infants had during the first year of life. The presence of Pf in peripheral blood samples was diagnosed by microscopy and by PCR. Pf genotyping was used to distinguish new from persistent infections and only new infections are represented in the figure. In the box and whisker plots: the horizontal lines of the boxes represent 25^th^, 50^th^ and 75^th^ percentiles; while the lower and upper limits of whiskers represent the 10^th^ and 90^th^ percentiles respectively; cross (+) shows the mean. Panels (a) and (c) show comparison between infants of PM− mothers and all infants of PM+ mothers. In panels (b) and (d), the infants in the PM+ group are subdivided into PM + Lo and PM + Hi. All 3 groups were compared using the Kruskal-Wallis test and the group-to-group comparisons were performed using the Mann-Whitney test. P values, where included, indicate significant differences. PCR, polymerase chain reaction; Pf, *Plasmodium falciparum*; PM, placental malaria.

Using PCR-based genotyping to distinguish between persistent and new infections, a total of 253 new infections were detected in the study cohort. Only new infections were included in the assessment of total number of times infants were infected. When all infants of PM+ mothers, irrespective of parasitemia, were compared with infants of PM− mothers, no significant differences were found (median [interquartile range] number of new infections detected by microscopy = 2.5 [1.0–4.8] for PM+ and 2.0 [0.3–3.8] for PM−, p = 0.2; and by PCR = 3.0 [2.0–5.8] for PM+ and 3.0 [2.0–5.0] for PM−, p = 0.3) (Fig. 3a,c). However, when PM+ infants were subdivided by placental parasitemia, PM + Lo infants had a higher number of new microscopic infections (3.5 [1.0–5.0]) than in PM− infants (2.0 [0.3–3.8], p = 0.05) (Fig. 3b). In addition, the number of new PCR-detected infections was significantly higher in the PM + Lo group (5.0 [3.0–7.0]) compared to the PM− group (3.0 [2.0–5.0], p = 0.02) and the PM + Hi group (3.0 [1.0–3.3], p = 0.009) (Fig. 3d). Normalizing the data of the number of times infants were infected with the number of samples collected from each infant during follow-up did not change the results. Together, the results show that the cumulative risk of Pf infection was highest in the PM + Lo group.

Collectively, the assessment of susceptibility in the infant cohorts demonstrated that low placental parasitemia at delivery was associated with increased susceptibility to malaria during the first year of life.

### Parasite burden of PM + Lo and PM + Hi mothers during pregnancy

**Figure 4:**
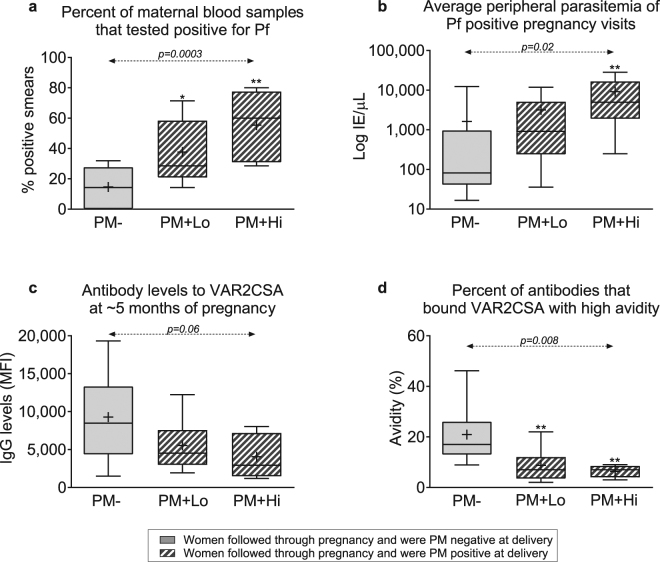
Relationship between PM at delivery with maternal Pf parasite load during pregnancy. Mothers of twenty-six infants (12 PM−, 9 PM + Lo and 5 PM + Hi) had been followed monthly during pregnancy. (**a**) Shows how often women were Pf-positive by microscopy during pregnancy normalized to the total number of prenatal monthly blood smears collected. (**b**) Average peripheral parasitemia of all positive visits. (**c**) The levels of IgG antibody to full-length VAR2CSA at 5 months of pregnancy. (**d**) The proportion of high avidity antibody to VAR2CSA. Data are shown as median and interquartile range (boxes) and 10^th^/90^th^ percentiles (whiskers). Crosses (+) within boxes represent means. P values over dashed lines represent level of significance of linear trend across ordered groups, from PM−, to PM + Lo to PM + Hi. Stars show significant two-way comparison of each PM+ group with respect to the PM− group: two stars (**), p < 0.01; one star (*), 0.01 < p < 0.05. IE, Pf-infected erythrocytes; PM, placental malaria.

To assess if differences in placental parasitemia at delivery reflected the mothers’ parasite load throughout pregnancy, monthly peripheral blood samples collected during the second and third trimesters of pregnancy were analyzed for 26 mothers of the above infants (12 PM−, 9 PM + Lo and 5 PM + Hi) (Fig. 4). The PM + Hi mothers experienced more Pf infections (median of 60% of monthly samples were positive for Pf) and higher median peripheral parasitemia (4,939 IE/µL) compared to PM + Lo (29% and 916 IE/µL) and PM− mothers (14% and 82 IE/µL) (Fig. 4a,b). In addition, VAR2CSA IgG that inhibits the binding of IE to placental villi and thus confers relative protection against PM^15–17^ were quantified in plasma collected at mid-pregnancy (~5 months) (Fig. 4c,d). A decrease in IgG levels and decrease in the percent of IgG that bound VARCSA with high avidity was observed across the groups: PM− (median IgG level [interquartile range] = 8,476 [4,363–13,333], percent of high avidity IgG = 17% [13-26]); PM + Lo (4,518 [2,979–7,582], 7% [3.5-12]); and PM + Hi (2,938 [1,483–7,186], 7% [4–8.5]). Lastly, 67% of PM + Lo women were multigravidae compared to only 20% of PM + Hi women.

### Parasite load in cord blood

Cord blood samples from 70 (31 PM−, 39 PM+) of the 80 infants originally enrolled were available to determine if placental parasitemia at delivery was associated with potential for fetal exposure to Pf *in utero*. The cord samples were examined for IE by microscopy, Pf-DNA by qPCR and Pf histidine-rich protein-2 (HRP2) by ELISA (Table 2). Only one case of congenital microscopic infection (154 IE/µL of cord blood) was identified and it was in the PM + Hi group. The proportion of newborns with Pf-DNA in cord blood was higher in the PM + Hi group (38.9%) than in the PM + Lo group (4.8%), p = 0.01. The percent of newborns positive for HRP2 in cord plasma and the HRP2 levels in positive newborns were slightly lower in the PM + Lo group than in the PM + Hi group but the difference was not significant. Collectively, the chance of fetal exposure to Pf and/or malarial antigens was higher in PM + Hi than PM + Lo pregnancies.

**Table 2:** Relationship between placental Pf parasitemia at birth and Pf products in cord blood. *Data are represented as median [min-max] of positive samples. Just one PM− newborn had trace amounts of Pf DNA and HRP2, thus P values only compare PM + Lo versus PM + Hi (Fisher’s exact test for proportions and Mann-Whitney test for medians). Note: All available cord blood samples were used including samples from 3 newborns, in the PM + Lo group, that were initially enrolled at birth but were not part of the postnatal follow-up cohort. n/a, not applicable; IE, *Plasmodium falciparum*-infected erythrocytes; HRP2, histidine-rich protein 2.

Analysis of umbilical cord blood	Placental parasitemia at delivery			P value
	None (PM−)	Low (PM + Lo)	High (PM + Hi)	
Number of newborns	31	21	18	n/a
Whole Pf parasites by microscopy				
Percent positive	0%	0%	5.9%	n/a
Parasite load, IE/µL	n/a	n/a	154	n/a
Pf DNA by qPCR				
Percent positive	3.2%	4.8%	38.9%	0.01
DNA concentration*, pg/mL	0.04	0.27	0.12 [0.02–3.01]	n/a
Pf HRP2 by ELISA				
Percent positive	3.2%	19.0%	22.2%	1.0
HRP2 concentration*, ng/mL	0.20	1.91 [0.37–6.88]	2.82 [0.15–5.59]	0.7

## Discussion

Studies have described associations between mothers having PM at delivery and an increased risk of their babies having Pf infections. This study focused on the relationship between placental parasite density at delivery and the susceptibility of infants to malaria. The results demonstrated for the first time that infants born to mothers with low, not high, placental parasitemia had a higher risk of malaria infection during the first year of life than infants born to mothers with no placental malaria at delivery. Infants in the PM + Lo group were more susceptible to Pf infection than all infants of PM+ mothers, indicating that much of the PM-associated increase in susceptibility was paradoxically accounted for by low placental parasitemia cases. The association between PM + Lo and susceptibility to Pf remained strong after adjusting for factors related to the environment (season of birth, residence within the village) and to malaria resistance (hemoglobin type, amount of transplacentally transferred anti-Pf antibodies). Although only a few cases of clinical malaria were diagnosed, presumably because anti-malarial treatment was administered each time asymptomatic microscopic infections were detected, the risk of clinical malaria also tended to be higher in infants of the PM + Lo group compared to the PM + Hi group.

PM + Lo mothers had lower overall parasite burden than PM + Hi mothers and were, therefore, less likely to have exposed their fetuses to malarial antigens. That is, women with low placental parasitemia at delivery were infected fewer times and had lower average peripheral blood parasitemia during the course of pregnancy compared to women with high placental parasitemia at delivery (Fig. 4). Also, more PM + Lo women were multigravidae (67%) than PM + Hi women (20%) and likely controlled their infections more efficiently since modeling of gravidity-dependent immunity to PM suggests that IE are more rapidly cleared from the placenta in multigravid than in primigravid women^14^. Furthermore, previous research has shown that high placental parasite load increases the chances of detecting parasites in cord blood^18^. In the current study, IE were detected in the cord blood of one newborn whose mother was PM + Hi and more newborns in the PM + Hi group had Pf-DNA in cord blood than newborns in the PM + Lo group, indicating higher prenatal Pf exposure in PM + Hi newborns. Interestingly, the presence of HRP2 in cord blood was similar between the PM + Hi and PM + Lo groups, possibly because HRP2 circulates for weeks after parasites and Pf DNA have been cleared^19^. Overall, mothers with low placental parasitemia at delivery had lower parasite load during pregnancy making it likely that their newborns had been exposed to lower amounts of Pf *in utero*.

The question of how exposure to Pf *in utero* programs the fetus for increased or reduced susceptibility to malaria is important. Peripheral tolerance to Pf antigens can be induced by T regulatory cells (T-regs) and may contribute to increased susceptibility. Fetal CD4^+^ T cells are biased towards T-regs since 15–20% of CD4^+^ T cells in fetal lymphoid organs are T-regs compared to <5% in adults^20,21^. Pf antigen exposure can induce the expansion of fetal T-regs which, in turn, suppresses antigen-driven responses by T effector cells^22–24^. On the other hand, some studies have shown strong T-helper 1 and T-helper 2 effector responses to Pf antigens in newborns of PM+ mothers, indicating that *in utero* Pf exposure can result in T cell sensitization^25^. Interestingly, a study in Kenya demonstrated T cell sensitization to Pf in a subset of newborns and T cell tolerance in other newborns within the same cohort^26^. The tolerant newborns had an approximately 2-fold increase in risk of malaria during the first three years of life compared to the sensitized newborns. However, the specific factors responsible for the dichotomy in fetal immune responses to Pf antigen remained elusive. Here, the data show that low placental parasitemia, in comparison with high placental parasitemia, is associated with increased risk of post-natal malaria and suggest that the outcome of sensitization or tolerance to Pf *in utero* may be influenced by the amount of Pf to which the fetus is exposed. The lack of cryopreserved cord blood cells in our cohort precluded any direct investigations of the link between placental parasite load and newborn T-cell sensitization/tolerance. Future studies are needed to identify the immune mechanism(s) induced *in utero* by fetal exposure to different amounts of antigens.

Data from animal studies provide clues on how the amount of antigen might skew human T cell responses towards tolerance or sensitization. For example, the antigen dose required to stimulate T-regs in mice is much lower than that needed for T effector cells to proliferate^27^. Thus, a relatively low Pf antigen concentration *in utero* might stimulate more T-regs than T effector cells leading to overall suppression of response to Pf. In addition, mouse transgenic T effector cells with receptors to OVA, proliferated very little in the presence of low OVA doses and T-regs, but overcame T-reg suppression when the dose of OVA was increased ~100 fold^28^. Therefore, high amounts of Pf may be required for human T effectors to escape T-reg suppression *in utero*.

The results from the current study demonstrate that placental parasitemia at delivery might be an indicator of fetal Pf exposure and potentially predict the susceptibility of infants to malaria. In this study, infants were dichotomized into two groups, i.e., below and above the median parasitemia which was 25 IE/µL of placental IVS blood. Clearly, additional studies are needed to determine if a universal cut-off parasitemia exists and if that threshold should be based on Pf parasite load in IVS blood smears, impression smears, or histological tissue sections. Nevertheless, infants of women who are Pf-negative by IVS blood smear but positive by histology or impression smears might potentially be at a high risk of malaria during infancy.

Even though the sample size of infants was relatively small, the current study replicated results from larger studies that have reported a shorter time to infection in infants born to PM+ compared to PM− women. Moreover, the study is unique in many ways. 1) Infections in infants were detected by PCR, in addition to microscopy, which significantly increased detection sensitivity and provided a better estimate of time to first infection. 2) The active monitoring of Pf infections is also reflected in the short interval between follow-up visits, ~1 month, in contrast to previous studies that monitored infants quarterly^6,29^ or biannually^26^. 3) New infections were distinguished from persistent infections by parasite genotyping thereby avoiding an overestimation of the number of infections. 4) Finally, maternal malaria was evaluated not just at delivery but also during pregnancy in a subset of mothers.

The translational implications of the findings are significant. If low placental parasitemia increases the risk of infant malaria, then malaria control interventions during pregnancy that do not eliminate Pf parasites from the placenta, but only reduce the Pf density, could increase the risk of malaria in the offspring. This is particularly important because there are reports from parts of Africa of decreasing efficacy of sulfadoxine-pyremethamine in eliminating parasites in pregnant women^30,31^. More effective interventions that eliminate Pf in pregnant women are therefore needed to protect both mothers and their offspring.

## Methods

### Study design

The study was conducted in the rural village of Ngali II, Cameroon, between January 2001 and May 2005. In this area, Pf transmission is perennial with two rainy (March–May, September–November) and two dry (December–February, June–August) seasons. The estimated entomological inoculation rate was 257 infective bites/person/year^13^. A total of 80 mother-newborn pairs were enrolled and the newborns were subsequently monitored during infancy. Early in the study, 8 mothers voluntarily withdrew their infants at ≤4 months of age, leaving 72 infants who were studied during the first year of life. Mothers of 26 infants had been enrolled at ≤14 weeks of pregnancy and followed monthly until delivery^13^; whereas, the other mother-baby pairs were enrolled at delivery. None of the mothers received intermittent preventive treatment for malaria in pregnancy (IPTp) since the study was performed before the implementation of IPTp in Cameroon^32,33^. The overall design of the current study is summarized in Fig. 1. Women in the study were not tested for HIV by the research team, but none of the women were reported to be infected by the local antenatal staff. Moreover, HIV prevalence in Cameroonian adults of child-bearing age in 2002 was ~5%^34^ and the rate of mother-to-child transmission of HIV in Cameroon has been estimated to be ~22.1%^35^. Thus the probability that a mother was HIV-positive and her baby was infected with HIV was low (i.e., 0.05 × 0.221 = ~1.1%).

During the first year of life, infants were seen at 8 time points corresponding to post-natal ages of 1.5, 3, 4, 5, 6, 8, 10 and 12 months. In addition, infants were monitored at home by four village health workers who arranged for sick children to be examined by the project physician. Therefore, each infant was actively monitored. None of the infants used bed nets and none received anti-malarial chemoprophylaxis within 2 weeks prior to each visit as reported by their parents. At all visits, the infant’s axillary temperature was measured and finger-prick blood collected. A clinical episode of malaria was defined as temperature > 37.5 °C plus blood-smear positive for Pf by microscopy. All infants with microscopic parasitemia regardless of body temperature were treated for malaria according to the Cameroonian government policy for treatment of childhood malaria. The study was approved by the institutional review board of Georgetown University and the National Ethics Committee of the Cameroonian Ministry of Public Health. Informed consent was obtained from the mothers of all infants and the study was carried out in accordance with relevant guidelines and regulations.

### Parasitological and hematological methods

Blood smears were made from maternal peripheral venipuncture samples collected monthly during pregnancy. The blood smears were stained with Diff-Quick (Baxter Scientific, IL) and examined for IE by microscopy. Pf parasitemia (IE/µL) was determined by counting the number of IE per 200 white blood cells (WBC) and multiplying by the woman’s WBC count.

At birth, about 2 mL placental IVS blood, 5 × 5 × 5 cm placental tissue biopsy and 5 mL of cord blood were collected. The IVS blood was obtained using the placental biopsy-pool method as previously described^36^. Briefly, the umbilical cord was quickly clamped at its placental insertion and a small tissue biopsy was made on the maternal side of the placenta, allowing IVS blood to pool at the excision site. The pooled IVS blood was aspirated into heparinized tubes. Cord blood was drawn directly from the umbilical vein. In previous studies, microsatelite DNA typing of paired IVS and cord blood samples collected using the same method did not show any significant admixture of maternal and fetal blood^25,37^. To determine parasitemia in placental IVS blood smears, the number of IE/µl was obtained by counting the number of IE per 200 IVS WBC and multiplying by the woman’s IVS WBC count. Furthermore, placental impression smears and histosections were made from placental biopsies and were semi-quantitatively examined for presence or absence Pf parasites. When IE were detected in histological sections or impression smears, but were not present in IVS blood smears, a parasitemia of < 14 IE/ µl (i.e., the lowest level detected in IVS blood smears) was assigned to the sample.

The amount of Pf DNA in cord blood was determined by nested quantitative PCR (qPCR), consisting of a conventional PCR step followed by qPCR. The assay was performed as previously reported^38^, with minor modifications: DNA was extracted from 100 µL of packed red cells premixed with 100 µL of PBS; and, GoTaq master mix (Promega, WI) and SsoAdvanced SYBR-Green Supermix (Bio-Rad, CA) were used for the PCR and qPCR steps, respectively. DNA from *in vitro* 3D7 parasites was quantified by Nanodrop and serially diluted (10 fold) to make standard curves.

During the first year of life, Pf infections were detected by microscopy and by nested PCR using finger-prick peripheral blood samples. DNA was extracted on spin columns using 200 µL of blood and the Pf 18 S ribosomal RNA gene was amplified^39,40^. Amplicons were electrophoresed through 1.5% agarose gel (5 V/cm) and SYBR-stained Pf bands were visualized. All Pf DNA-positive samples were evaluated for polymorphisms in the K1, MAD20 and R033 allelic families of Pf MSP1 block 2-4, and in the FC27 and 3D7 allelic families of Pf MSP2^13,40^. An infant was considered to have a “new infection” if one or more Pf alleles were detected that had not been present at the preceding visit. Finally, standard strip-based electrophoresis was used for hemoglobin (HbAA and HbAS) genotyping using infant peripheral blood samples.

### Immunological methods

To assess the degree of protection from PM during pregnancy, the levels of IgG and proportion of high avidity IgG antibodies to full-length VAR2CSA at ~5 months of pregnancy were measured as previously described^17^.

To determine the level of anti-Pf antibodies that was passively acquired by infants *in utero*, cord blood was tested for IgG to Pf MSP1_42_ (FVO strain) and EBA175 antigens by Luminex assay. Briefly, 1 µg of each recombinant antigen was coupled to 1 million beads with a different spectral address per antigen^25^ and then 50 µL of beads (i.e. 3000 beads for each antigen) was mixed with 50 µL of diluted plasma (1:100 in PBS-1%BSA) and incubated for 1 h on a shaker at room temperature. After two washes with PBS-0.05% Tween and one wash with PBS-1%BSA, 100 µL of 1:250 dilution of 2 µg/ml phycoerythrin-conjugated goat anti-human IgG was added to each well and incubated for 1 h. Beads were washed, resuspended in 100 µL of PBS-1%BSA and analyzed in the LiquiChip 200 analyzer. Results are reported as median fluorescence intensity (MFI).

To determine if the Pf antigen HRP2 was present in cord blood, samples were screened using the CELISA kit (Cellabs, Australia). Whole blood was prepared by recombining equal volumes of cyropreserved plasma and packed red blood cells. The assay was conducted in duplicate using 100ul of blood per well following the manufacturer’s instructions. Serial dilutions of a positive-control sample provided in the kit were used to generate standard curves and the cut-off for positivity were established as specified by the manufacturer.

### Statistical methods

Kaplan-Meier survival curves and log-rank tests were used to compare the median time to first infection among infant study groups. Right-censoring was done either at withdrawal from study, death, age 1 year, or at end of study period, whichever occurred first. Cox regression was used to assess hazard ratios and 95% CI. Hazard ratios were adjusted for covariates that could potentially influence susceptibility to infection, including gravidity^3,5^; residence and season at birth^13,41^; HbAA versus HbAS^42^; and cord plasma IgG to Pf merozoite antigens MSP1 and EBA175^43,44^. The cord Pf IgG levels were stratified into 3 categories: > 67^th^ percentile, 33^rd^ to 66^th^ percentile and < 33^rd^ percentile. Ranking was done because unit changes in Pf IgG median fluorescence intensity (range: 500 to 24,000 MFI) were not expected to have significant effects.

Other data were presented as percentages or as medians and interquartile ranges, unless otherwise stated. Fisher’s exact test was used to compare categorical data. Because many continuous variables were not normally distributed (D’Agostino-Pearson omnibus normality test) and owing to the small sample size in some sub-analyses, non-parametric tests were used for group comparisons. The Kruskal-Wallis test was used to compare changes in variables such as average number times infants in the 3 study groups were infected while Mann-Whitney test was used for group-to-group comparisons. Where appropriate, a linear trend test for changes across groups with increasing parasitemia (i.e., PM−, PM + Lo and PM + Hi in that order) was applied. P values were expressed to the nearest non-zero number after decimal point and P < 0.05 was considered significant. All P values were two-tailed.

### Data availability

The datasets generated during and/or analyzed during the current study are available from the corresponding author on reasonable request.

## Electronic supplementary material

Supplementary Figure

## References

[1] Fried M, Duffy PE (1998). Maternal malaria and parasite adhesion. J. Mol. Med. (Berl)..

[2] Guyatt, H. L. & Snow, R. W. Impact of malaria during pregnancy on low birth weight in sub-Saharan Africa. *Clin*. *Microbiol*. *Rev*. **17**, 760–769, table of contents (2004). 10.1128/CMR.17.4.760-769.2004 PMC523568 15489346

[3] Mutabingwa TK (2005). Maternal Malaria and Gravidity Interact to Modify Infant Susceptibility to Malaria. PLoS Med..

[4] Le Port A (2011). Infections in infants during the first 12 months of life: role of placental malaria and environmental factors. PLoS One.

[5] Schwarz NG (2008). Placental malaria increases malaria risk in the first 30 months of life. Clin. Infect. Dis..

[6] Sylvester B (2016). Prenatal exposure to Plasmodium falciparum increases frequency and shortens time from birth to first clinical malaria episodes during the first two years of life: prospective birth cohort study. Malar. J..

[7] Le Hesran JY (1997). Maternal placental infection with Plasmodium falciparum and malaria morbidity during the first 2 years of life. Am. J. Epidemiol..

[8] Accrombessi, M. *et al*. Malaria in pregnancy is a predictor of infant haemoglobin concentrations during the first year of life in Benin, West Africa. *PLoS One* **10** (2015). 10.1371/journal.pone.0129510 PMC4460073 26052704

[9] Bardají A (2011). Impact of malaria at the end of pregnancy on infant mortality and morbidity. J. Infect. Dis..

[10] De Beaudrap P (2016). Timing of malaria in pregnancy and impact on infant growth and morbidity: a cohort study in Uganda. Malar. J..

[11] Moya-Alvarez V, Abellana R, Cot M (2014). Pregnancy-associated malaria and malaria in infants: an old problem with present consequences. Malar. J..

[12] Brabin BJ (1983). An analysis of malaria in pregnancy in Africa. Bull. World Health Organ..

[13] Leke RFG (2010). Longitudinal studies of Plasmodium falciparum malaria in pregnant women living in a rural Cameroonian village with high perennial transmission. Am. J. Trop. Med. Hyg..

[14] Walker PGT (2013). A model of parity-dependent immunity to placental malaria. Nat. Commun..

[15] Agbor-Enoh ST (2003). Chondroitin sulfate proteoglycan expression and binding of Plasmodium falciparum-infected erythrocytes in the human placenta during pregnancy. Infect. Immun..

[16] Rogerson SJ, Hviid L, Duffy PE, Leke RF, Taylor DW (2007). Malaria in pregnancy: pathogenesis and immunity. Lancet Infectious Diseases.

[17] Tutterrow, Y. Lo *et al*. High Avidity Antibodies to full-length VAR2CSA correlate with absence of Placental malaria. *PLoS One* **7** (2012). 10.1371/journal.pone.0040049 PMC3383675 22761948

[18] Redd SC, Wirima JJ, Steketee RW, Breman JG, Heymann DL (1996). Transplacental transmission of plasmodium falciparum in Rural Malawi. Am. J. Trop. Med. Hyg..

[19] Iqbal J, Siddique A, Jameel M, Hira PR (2004). Persistent histidine-rich protein 2, parasite lactate dehydrogenase, and panmalarial antigen reactivity after clearance of Plasmodium falciparum monoinfection. J. Clin. Microbiol..

[20] Mold JE, Anderson CC (2013). A discussion of immune tolerance and the layered immune system hypothesis. Chimerism.

[21] Burt TD (2013). Fetal Regulatory T Cells and Peripheral Immune Tolerance In Utero: Implications for Development and Disease. American Journal of Reproductive Immunology.

[22] Mackroth MS (2011). Human cord blood CD4 + CD25hi regulatory T cells suppress prenatally acquired T cell responses to Plasmodium falciparum antigens. J. Immunol..

[23] Brustoski K (2006). Reduced cord blood immune effector-cell responsiveness mediated by CD4 + cells induced in utero as a consequence of placental Plasmodium falciparum infection. J. Infect. Dis..

[24] Flanagan KL (2010). The effect of placental malaria infection on cord blood and maternal immunoregulatory responses at birth. Eur. J. Immunol..

[25] Metenou, S., Suguitan, A. L., Long, C., Leke, R. G. F. & Taylor, D. W. Fetal immune responses to Plasmodium falciparum antigens in a malaria-endemic region of Cameroon. The Journal of Immunology **178** (2007). 10.4049/jimmunol.178.5.2770 17312120

[26] Malhotra, I. *et al*. Can prenatal malaria exposure produce an immune tolerant phenotype?: A prospective birth cohort study in Kenya. *PLoS Med*. **6** (2009). 10.1371/journal.pmed.1000116 PMC2707618 19636353

[27] Takahashi T (1998). Immunologic self-tolerance maintained by CD25 + CD4 + naturally anergic and suppressive T cells: induction of autoimmune disease by breaking their anergic/suppressive state. Int. Immunol..

[28] George TC, Bilsborough J, Viney JL, Norment AM (2003). High antigen dose and activated dendritic cells enable Th cells to escape regulatory T cell-mediated suppression *in vitro*. European Journal of Immunology.

[29] Boudová S (2017). Placental but not peripheral Plasmodium falciparum infection during pregnancy is associated with increased risk of malaria in infancy. J. Infect. Dis..

[30] Desai M (2016). Impact of sulfadoxine-pyrimethamine resistance on effectiveness of intermittent preventive therapy for Malaria in pregnancy at clearing infections and preventing low birth weight. Clin. Infect. Dis..

[31] Walker PGT, Floyd J, Ter Kuile F, Cairns M (2017). Estimated impact on birth weight of scaling up intermittent preventive treatment of malaria in pregnancy given sulphadoxine- pyrimethamine resistance in Africa: A mathematical model. PLoS Med..

[32] World Health Organization. *A strategic framework for malaria prevention and control during pregnancy in the African region*. (2004).

[33] Leke RGF, Taylor DW (2011). The Use of Intermittent Preventive Treatment With Sulfadoxine-Pyrimethamine for Preventing Malaria in Pregnant Women. Clin Infect Dis..

[34] National AIDS Control Committe- Central Technical Group (NACC-CTG). The Impact of HIV and AIDS in Cameroon through 2020 (2010).

[35] Nguefack HLN (2015). Estimating mother-to-child HIV transmission rates in Cameroon in 2011: a computer simulation approach. BMC Infect. Dis..

[36] Suguitan AL (2003). Malaria-associated cytokine changes in the placenta of women with pre-term deliveries in Yaounde, Cameroon. Am. J. Trop. Med. Hyg..

[37] Tassi Yunga, S. *et al*. Timing of the human prenatal antibody response to Plasmodium falciparum antigens. *PLoS One* **12** (2017). 10.1371/journal.pone.0184571 PMC5614534 28950009

[38] Tran TM (2014). A nested real-time PCR assay for the quantification of Plasmodium falciparum DNA extracted from dried blood spots. Malar. J..

[39] Snounou G, Viriyakosol S, Jarra W, Thaithong S, Brown KN (1993). Identification of the four human malaria parasite species in field samples by the polymerase chain reaction and detection of a high prevalence of mixed infections. Mol. Biochem. Parasitol..

[40] Walker-Abbey A (2005). Malaria in pregnant Cameroonian women: the effect of age and gravidity on submicroscopic and mixed-species infections and multiple parasite genotypes. Am. J. Trop. Med. Hyg..

[41] Apinjoh, T. O. *et al*. Determinants of infant susceptibility to malaria during the first year of life in south western Cameroon. *Open Forum Infect*. *Dis*. **2** (2015). 10.1093/ofid/ofv012 PMC4438893 26034763

[42] Gong L, Parikh S, Rosenthal PJ, Greenhouse B (2013). Biochemical and immunological mechanisms by which sickle cell trait protects against malaria. Malar. J..

[43] Hogh B, Marbiah NT, Burghaus PA, Andersen PK (1995). Relationship between maternally derived anti-Plasmodium falciparum antibodies and risk of infection and disease in infants living in an area of Liberia, West Africa, in which malaria is highly endemic. Infect. Immun..

[44] Branch O (1998). A longitudinal investigation of IgG and IgM antibody responses to the merozoite surface protein-1 19-kiloDalton domain of Plasmodium falciparum in pregnant women and infants: associations with febrile illness, parasitemia, and anemia. Am. J. Trop. Med. Hyg..

