# Supplementary material for: Increased Susceptibility to Plasmodium falciparum in Infants is associated with Low, not High, Placental Malaria Parasitemia
Source: Sci Rep. 2018 Jan 9;8:169. doi: 10.1038/s41598-017-18574-6 (PMC5760570; doi:10.1038/s41598-017-18574-6)
Supplement: Supplementary file 1 — Supplementary Figure [file 41598_2017_18574_MOESM1_ESM.doc]

**Increased Susceptibility to *Plasmodium falciparum* in Infants is associated with Low, not High, Placental Malaria Parasitemia**

**Samuel Tassi Yunga^1^, Genevieve G. Fouda^2^, Grace Sama^3^, Julia B. Ngu^4^, Rose G.F. Leke^3^, Diane W. Taylor^1*^**


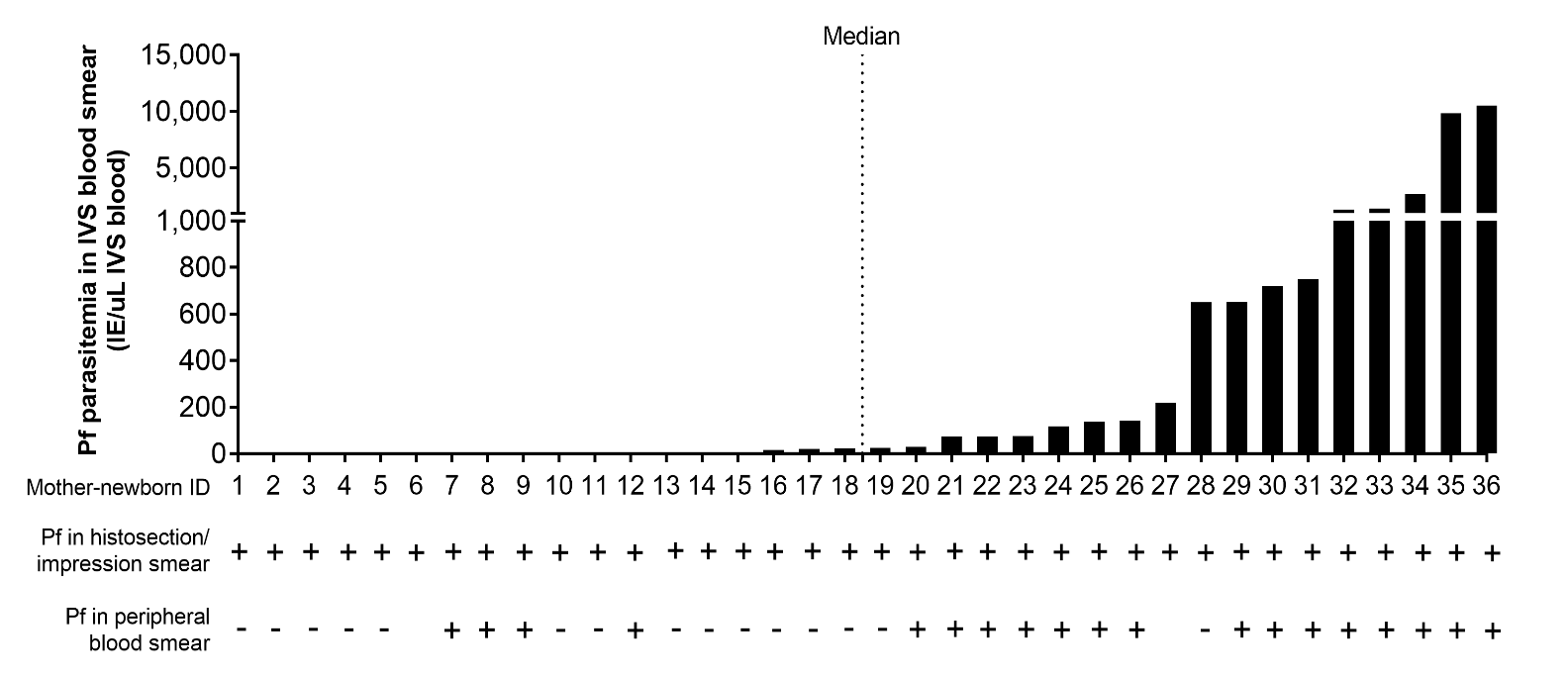


**Supplementary Figure S1**: Distribution of placental parasitemia in PM+ cases. Pf parasitemia was quantified in smears of placental intervillous space (IVS) blood. For mother-newbon pairs 1-15, Pf were detected in placental histosections or impression smears but not in IVS smear. The presence of Pf in the placenta (IVS, histosection or impression smear) was not always accompanied by Pf in peripheral blood, suggesting placental sequestration of Pf. (+) and (–) indicate positive and negative for Pf respectively (space = data not available)
